# Thymus-derived hormonal and cellular control of cancer

**DOI:** 10.3389/fendo.2023.1168186

**Published:** 2023-07-17

**Authors:** Wilson Savino, Ailin Lepletier

**Affiliations:** ^1^ Laboratory on Thymus Research, Oswaldo Cruz Institute, Oswaldo Cruz Foundation, Rio de Janeiro, Brazil; ^2^ Brazilian National Institute of Science and Technology on Neuroimmunomodulation, Oswaldo Cruz Institute, Oswaldo Cruz Foundation, Rio de Janeiro, Brazil; ^3^ Rio de Janeiro Research Network on Neuroinflammation, Oswaldo Cruz Institute, Oswaldo Cruz Foundation, Rio de Janeiro, Brazil; ^4^ INOVA-IOC Network on Neuroimmunomodulation, Oswaldo Cruz Institute, Oswaldo Cruz Foundation, Rio de Janeiro, Brazil; ^5^ Institute for Glycomics, Griffith University, Gold Coast, QLD, Australia

**Keywords:** thymus, thymus-derived peptide hormones, T cell subsets, immunoendocrine interactions, cancer therapy

## Abstract

The thymus gland is a central lymphoid organ in which developing T cell precursors, known as thymocytes, undergo differentiation into distinct type of mature T cells, ultimately migrating to the periphery where they exert specialized effector functions and orchestrate the immune responses against tumor cells, pathogens and self-antigens. The mechanisms supporting intrathymic T cell differentiation are pleiotropically regulated by thymic peptide hormones and cytokines produced by stromal cells in the thymic microenvironment and developing thymocytes. Interestingly, in the same way as T cells, thymic hormones (herein exemplified by thymosin, thymulin and thymopoietin), can circulate to impact immune cells and other cellular components in the periphery. Evidence on how thymic function influences tumor cell biology and response of patients with cancer to therapies remains unsatisfactory, although there has been some improvement in the knowledge provided by recent studies. Herein, we summarize research progression in the field of thymus-mediated immunoendocrine control of cancer, providing insights into how manipulation of the thymic microenvironment can influence treatment outcomes, including clinical responses and adverse effects of therapies. We review data obtained from clinical and preclinical cancer research to evidence the complexity of immunoendocrine interactions underpinning anti-tumor immunity.

## 1 Introduction

**Figure 1:**
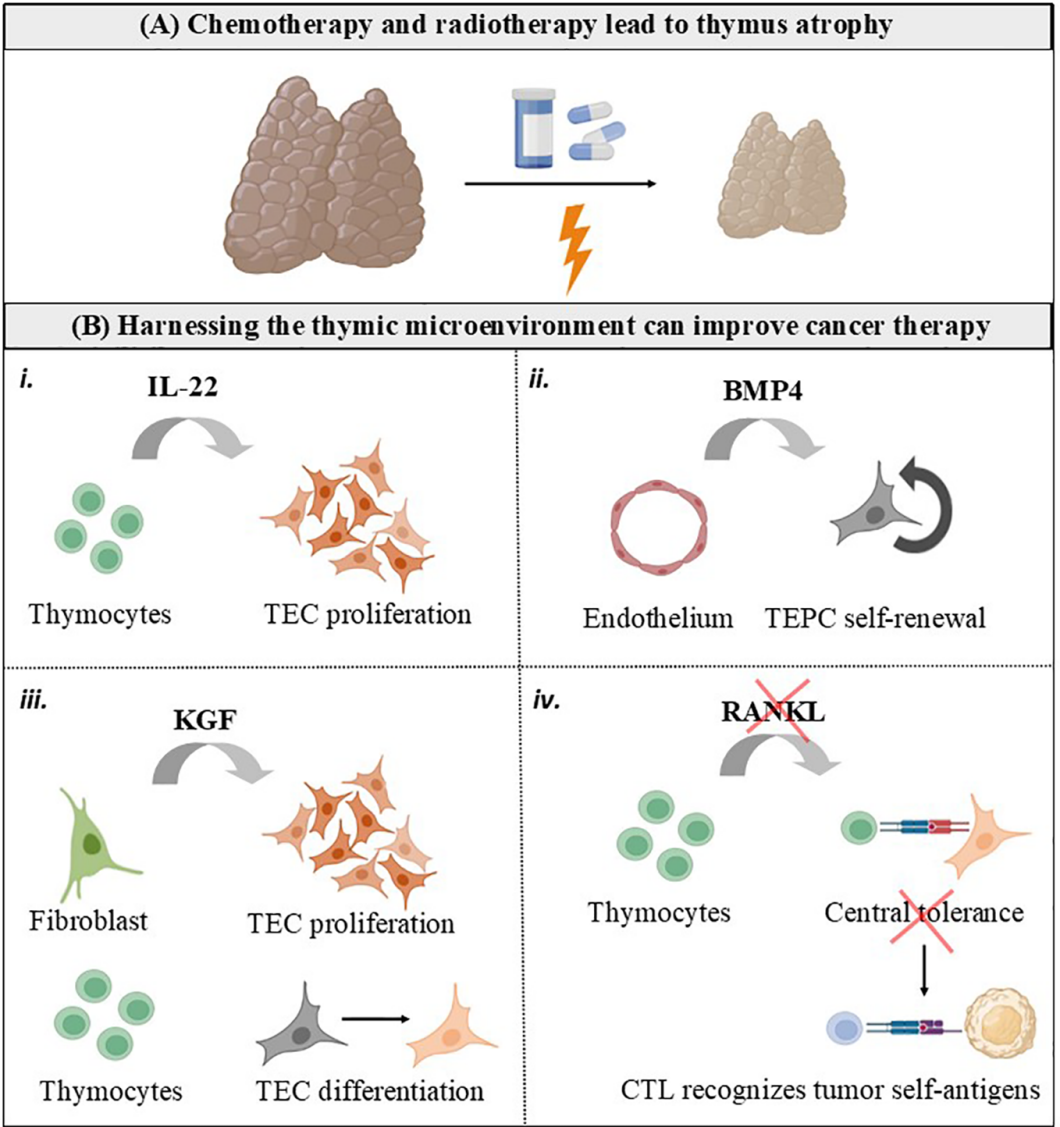
Harnessing the thymic microenvironment to improve cancer therapy. **(A)** Conventional cancer therapies, including chemotherapy and radiotherapy, causes the thymus to transiently involute due to loss of TEC and thymocytes. **(B)** Cytokines and growth factors produced by the thymic microenvironment can be used as adjuvants for cancer therapy. *i.* IL-22 is produced intrathymically by T cells and drive thymic regeneration following radiation damage, by inducing TEC proliferation. *ii.* BMP4 is produced by thymic endothelial cells in response to radiation and stimulate self-renewal of TEC progenitors (TEPC). *iii.* KGF is produced by thymic fibroblasts in the fetus and by mature thymocytes in the adult thymus. KGF has an important role in repairing epithelial tissues, inducing TEC proliferation and differentiation. *iv*. RANKL is mainly provided by positively selected CD4^+^ thymocytes and innate lymphoid cells, and controls central tolerance by inducing mTEC proliferation, differentiation, and regeneration. It is possible that RANKL blockade interrupts central tolerance and unleashes cytotoxic T cells (CTL) possessing TCR with self-reactive features to recognize tumor cells expressing self-antigens.

T lymphocytes (T cells) are critical orchestrators of the adaptive immune response that optimally eliminates tumor cells. The thymus is uniquely committed to T cell production by providing an inductive microenvironment in which bone marrow-derived progenitors undergo proliferation, T cell receptor (TCR) gene rearrangements and differentiation into mature T cells. Normal thymic architecture is essential for the proper development of T cells, which is mediated by interactions between resident thymic T cells (thymocytes) and thymic epithelial cells (TEC). TEC expressing the autoimmune regulator (AIRE) maintain immune central expressing the autoimmune regulator (AIRE) to maintain immune central tolerance by guiding the clonal deletion of autoreactive thymocytes and development of regulatory T cells (Treg) (1). Mature T cells egress from the thymus and enter the bloodstream, participating as a central component of the adaptive immune system as mediators of anti-tumor immunity (2, 3). Accordingly, therapies harnessing cytotoxic T cells have markedly improved the care of patients with multiple types of cancer (3, 4). Different from immunotherapies based on adoptive T cell therapy and immune-checkpoint inhibitors (ICI, designed to target the immune-inhibitory receptors CTLA-4, PD1 and TIGIT on T cells), conventional which are designed to target the immune-inhibitory receptors CTLA-4, PD1 and TIGIT on T cells, conventional cyto-ablative cancer therapies (i.e. chemotherapy and radiotherapy) directly target the tumor cell. These conventional cancer therapies lead to damage of the thymic structure, with significant impairment of mature TEC generation and the naïve T cell repertoire, a hallmark of thymus atrophy (**Figure 1A**) (5, 6).

In comparison to T cells, the role of thymic hormones in controlling tumor progression is understudied. Thymic peptide hormones, including thymosins, thymulin and thymopoietin, are secreted by TEC and can either act locally on thymocytes or circulate in the periphery to impact the differentiation and function of T cells and other immune subsets (1, 7, 8). Purified peptides from the thymus as well as thymic peptide analogues and cytokines have been trialled in several studies investigating their impact on the response and tolerability of standard chemotherapy, radiotherapy, or both (9, 10). As such, revealing the relationship between thymic hormones/cytokines, T cells and tumor cells may lead to novel strategies to improve the outcome of patients with cancer.

## 2 Intrathymic T cell differentiation

The thymus, a primary lymphoid organ where differentiation of T cells take place, is a source of a variety of soluble immune-related moieties, including cytokines, chemokines, classic hormones, and neurotransmitters. Additionally, key interactions occur through cell-cell and cell-matrix interactions. Intrathymic T cell development has been extensively reviewed in the last few years by several research groups (11–15). During intrathymic T cell differentiation, bone marrow-derided T cell precursors enter the thymus through blood vessels at the corticomedullary junction where they encounter the thymic microenvironment. The thymic tridimensional network is constituted of cellular components, such as TEC, thymic dendritic cells, macrophages and fibroblasts, as well as secreted non-soluble and soluble molecules such as the extracellular matrix proteins (including among other fibronectins and laminins), cytokines (as interleukin (IL)-2, IL-6, IL-7, IL-22 and RANKL), chemokines (CXCL12, CCL4, CCL7, CCL19 and CCL25), thymic hormones (as thymosin, thymopoietin, and thymulin), growth factors (as BMP4, KGF and FLT3) and different typical soluble components of nervous tissues, including as neuropeptides and neurotransmitters.

The thymus is histologically divided in lobules, each one comprising two main regions: the cortex and the medulla. Immature CD4^-^CD8^-^ double-negative (DN) and CD4^+^CD8^+^ double-positive (DP) thymocytes are in the cortex, whereas mature CD4^+^ or CD8^+^ single-positive (SP) thymocytes are in the medulla. In physiological conditions, these SP cells leave the thymus to colonize the T-dependent regions of secondary lymphoid organs. This process is under the control of the thymic microenvironment. During intrathymic T cell development, thymocytes are exposed to interactions involving the TCR and major histocompatibility complex (MHC) proteins expressed by TEC and dendritic cells. TCR-expressing DP developing thymocytes are rescued from programmed cell death during positive selection by interaction with self-antigens presented to the TCR by MHC molecules. Those thymocytes that were positively selected move towards the medulla to interact with self-antigens presented by AIRE-expressing medullary TEC (mTEC), being thus tested for negative selection. If this is the case, differentiating thymocytes will undergo apoptosis, due to high avidity interaction of the TCR with self-antigens presented by the MHC class I or class II molecules expressed by microenvironmental cells.

Positioning of developing thymocytes to specialized thymic microdomains depend on multiple interactions including cell-cell and cytokine/chemokine-mediated interactions. For example, CXCL12 is secreted by TEC, and preferentially attracts immature DN and DP cells, by ligation with the receptor CXCR4. The chemokine CCL25 also attracts immature thymocytes and its receptor, CCR9, is expressed at all stages of murine thymocyte differentiation. Notably, alike the thymic microenvironment that guide the development of thymocytes, reciprocally thymocytes control the differentiation and organization of TEC (**Figure 1B**), a process commonly known as thymic cross-talk (16, 17). One of the classical examples of this bidirectional interaction between thymocytes and the thymic stroma is provided by the interactions between thymocytes, mTEC and dentric cells, which result in deletion of autoreactive T cells and the generation of natural regulatory T cells at the same time that the developing thymocytes control the composition and complex three-dimensional organization of the thymic medulla (16).

**Figure 2:**
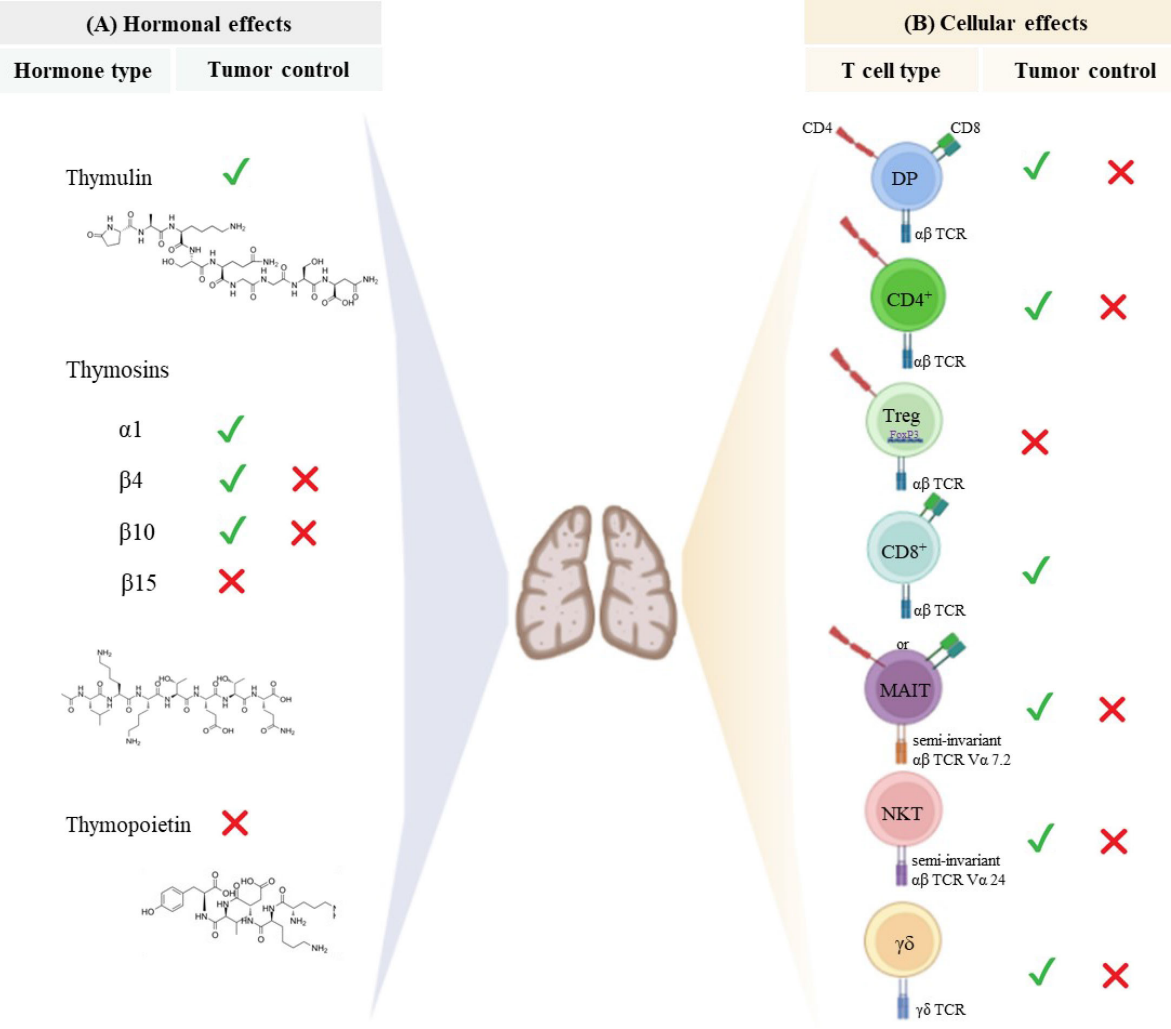
Thymus-derived peptides and immune cells regulate tumor growth. **(A)** Thymic peptide hormones are synthesized within the thymus and exert important regulatory effects on tumor cells. Thymosin α1 can induce anti-tumor immune responses and directly act on tumor cells to retrain tumor growth. Although β-thymosins (herein exemplified by β4, β10 and β15) can facilitate tumor progression, the β4 and β10 isoforms have also been reported to present suppressive tumor effect is some disease settings. Despite the paucity of available data, thymulin appears to promote anti-tumor effects, while high levels of thymopoietin have been associated with tumor cell proliferation and survival. The chemical structure of synthetic thymosin β4, thymulin and thymopoietin is shown. **(B)** The thymus provides the physiological microenvironment critical for the development of different types of T cells. It is possible that immature T cells expressing both CD4 and CD8 co-receptors can migrate to the immune periphery exerting either cytotoxic or immunosuppressive effects depending on the disease context. Similarly, conventional CD4^+^ T cells and unconventional T cells (MAIT, NKT and γδ T cells) can exert dual effect on tumor cells. Cytotoxic CD8 ^+^ T cells are the main effector cells to promote anti-tumor immunity, while regulatory T cells expressing both CD4 and the transcription factor Foxp3 have been largely investigated for its immunosuppressive properties and ability to hamper effective anti-tumor immune responses. The signals represent the role of hormones and T cells in controlling (✓) or inducing (✗) tumor progression.

Overall, in physiological conditions, mature CD4^+^ and CD8^+^ SP thymocytes exit the thymus to populate the peripheral lymphoid organs and participate in adaptive immune responses, including those underpinning anti-tumor immunity. Of note, thymic-derived hormones, cytokines, and growth factors can be manipulated to improve responses to cancer therapy, as summarized in **Figures 1B**, **2**.

## 3 Thymic hormones affect tumor cell biology *via* direct and immune-related mechanisms

In addition to the above cited molecules secreted intrathymically, we can find the thymic peptides, being considered as part of a heterogenous family of polypeptide hormones synthesized within the thymus. Studies with purified native thymic peptides and analogue peptides have shown a variety of regulatory effects on oncogenic diseases, mediated both by interactions with the host’s immune compartment (18, 19) and direct interaction with tumor cells (20–22). The effect of the main thymic peptides able to modulate tumor progression is summarized in **Figure 2A** and described below.

### 3.1 Thymosins

Includes two major families termed as α and β, which are classically regarded as main regulators of intrathymic T cell differentiation (1, 23). Besides, thymosins can also impact other immune cell types and tumor cells.

#### 3.1.1 Thymosin α1 (Tα1)

Tα1 represents a varied range of targets for its immune-enhancing activity and has shown promising results in improving immune responses in different types of malignancies (10). Tα1 increases the number of tumor-infiltrating CD4^+^ and CD8^+^ T cells in melanoma and breast cancer xenograft models (24) due to its role in induction of T cell differentiation, enhancement of IFN-γ and IL-2 production, and downregulation of T cell apoptosis (25, 26). Besides its effect on T cells, Tα1 has been reported to accelerate the replenishment and maturation of macrophages in the bone marrow of mice severely damaged by the chemotherapy (27) and block the intratumoral accumulation of myeloid suppressor cells in a mouse subcutaneous xenograft tumor model (28). Besides modulating the host immune system, Tα1 can act directly on tumor cells, exhibiting the ability to restrain tumor growth by its proapoptotic and anti-proliferative properties demonstrated in human leukemia cells lines (20) and preclinical cancer models (29), including prevention of tumor progression in immunosuppressed mice (30, 31).

#### 3.1.2 Thymosin β4 (Tβ4)

It is the most abundant thymic hormone among the thymosin family, with regenerative and anti-inflammatory properties, being expressed not only in the thymus but also in many other tissues (32). Tβ4-derived synthetic peptides have been shown to induce the angiogenesis, invasion and metastasis of melanoma tumor cells when administrated *in vivo* (21). Corroborating with its pro-tumorigenic role, Tβ4 gene silencing suppressed proliferation and invasion of NSCLC cells (22). Inversely, decreased expression of this hormone has been associated with poor prognosis in patients with multiple myeloma (20), while overexpression of Tβ4 led to decreased proliferative and migratory capacities of tumor cells in a mouse model of multiple myeloma (33). These data evidence a dual role of Tβ4 in cancer, either promoting or inhibiting tumor progression.

#### 3.1.3 Thymosin β10

Thymosin β10 has multiple pro-tumorigenic roles. Its overexpression correlates with disease progression in bladder cancer (34), hepatocellular carcinoma (35), NSCLC (36), and pancreatic cancer (37). Thymosin β10 has been shown to promote tumor-associated macrophage conversion into immunosuppressive M2 types and favor progression of lung adenocarcinoma (38). On the other hand, administration of thymosin β10 to a xenograft model of human ovarian cancer inhibited tumor cell invasion and metastasis (39) and induced a high rate of apoptosis in ovarian cancer cell lines (40).

#### 3.1.4 Thymosin β15

It has been pointed as a novel regulator of tumor cell motility and is upregulated in metastatic prostate cancer (41). In addition, high expression of thymosin β15 has been correlated with the metastatic potential of mouse lung carcinoma and human breast carcinoma cells (42).

Therefore, while Tα1 presents vast anti-tumors effects and have been shown synergic effects with multiple anti-cancer therapies (reviewed in section 7), the members of the beta thymosin family can either stimulate or inhibit tumor progression, differentially interfering with prognosis according to the type of cancer.

### 3.2 Thymopoietin

It is a thymus-derived polypeptide involved in the modulation of tumor cell biology. Studies conducted in gastric cancer patients showed that high levels of thymopoietin were associated with a significantly poorer overall survival, and that knockdown of this hormone suppressed gastric cancer and glioblastoma cell proliferation and survival (43, 44). In pancreatic cancer cells, knockdown of thymopoietin not only inhibited cell proliferation but also suppressed migration, invasion, and metastasis (45). Accordingly, long non-coding thymopoietin RNA 1 (TMPO-AS1) is highly expressed in hepatocellular carcinoma cells and promotes tumor development by enhancing cell viability, proliferation, and stemness and inhibiting apoptosis (46). Besides its contribution to tumor cell expansion and metastasis thymopoietin induced non‐significant reduction in the number of patients experiencing neutropenia during chemotherapy (47). Overall, the scarce data on the role of thymopoietin in cancer outcome remains to be better defined.

### 3.3 Thymulin

The thymic hormone thymulin is a zinc-containing nonapeptide specifically produced by TEC, which can be simultaneously detected with Tα1 and thymopoietin in the epithelial network of the human thymus (48, 49). A non-exhaustive list of thymulin biological functions can be seen in **Box 1**.

**Box 1 Biological Functions of the Thymic Hormone thymulin.**

- Increase of CD3, CD4 and CD8 expression in immunodeficient children
- Upregulation of NK activity in humans and mice
- Stimulation and activation of mouse intraepithelial T lymphocytes
- Increase in IL-2 production by normal mouse thymocytes and nude mouse splenocytes
- Increase in IL-1 and decrease in IL-6 and TNF-α production by peripheral blood mononuclear cells from normal volunteers
- Increase in IgA and IgE synthesis in patients with ataxia telangectasia
- Increase of LPS-induced polyclonal B cell responses of CBA/N mice
- Enhancement of T cell-dependent macrophage-mediated killing of microorganisms
- Delayed skin graft rejection time in normal mice
- Reduction in anti-DNA antibody production and glomerulonephritis in mice undergoing lúpus erithematosus
- *In vitro* modulation of T cell markers in human rheumatoid arthritis and systemic lupus erythematosus patients,
- *In vivo* prevention of encephalomyocarditis virus-induced diabetes and myocarditis in mice
- Decrease in hind paw swelling and anti-type II collagen antibody production in experimental arthritis in rats
- Protection against chronic septic inflammation in mice
- Anti-inflammatory and analgesic action in neuropathic pain
- Therapeutic reversion of asthma-induced pathology in the respiratory tract
*Based on references (1, 50, 51)

The role of thymulin in cancer is poorly understood and the data available so far suggest that thymulin can induce anti-tumor immunity and inhibits proliferation of tumor cells. An early study investigating young patients affected by acute lymphoblastic leukemia demonstrated a positive correlation between the levels of zinc-bound active thymulin and lymphocyte proliferative responses to mitogens, at the same time the proliferation rate of human lymphoblastoid cells reduced when active thymulin was added to the cultures (52). Interestingly, we have previously identified thymus atrophy in a mouse model of lung cell carcinoma, which was abolished after treatment with zinc chloride (a stimulator of thymulin secretion) (53), indicating that thymulin and tumor cells may play a two-way regulatory role in the thymic microenvironment.

## 4 Thymus-derived immune cells play a complex role in cancer, either promoting or inhibiting tumor progression

T cells are regarded as the main players in tumor immunity. The thymus generates naïve T cells able to differentiate into populations of effector and memory T cells, providing long-lasting immune responses to diverse tumor antigens. Among the immune cells that contribute to anti-tumoral responses, conventional T cells (T cells that express an αβ T cell receptor, as well as a co-receptor CD4 or CD8), have attracted the attention of tumor immunologists and clinical scientists. These cells present highly diverse TCR, each composed of a heterodimeric αβ chain, to recognize processed antigenic peptide presented by MHC molecules on other cells (**Figure 1B**). Besides conventional T cells, other types of T lymphocytes also developed in the thymus, such as Treg and innate-like unconventional T cells (including MAIT, NKT and γδ T cells), orchestrate the immune responses against tumor cells (54, 55), as summarized in **Figure 2B** and reviewed below.

### 4.1 CD8^+^ T cells

Cytotoxic T cells expressing cell-surface CD8 are the most powerful effectors in the anti-tumor immunity and constitute a critical determinant of response to cancer immunotherapies (2, 56). Once conjugated to a tumor cell, secretory granules in the CD8^+^ T cell cytoplasm traffic to the immunological synapse and release a cargo of deadly cytotoxic proteins (mostly represented by perforin and granzymes) leading to membrane damage, induction of reactive oxygen species, nuclear envelope rupture and DNA damage, and resulting in tumor cell death (57). Besides cytotoxic events unleashed by direct interaction with tumor cells, the protective role of CD8^+^ T cells in the tumor microenvironment is associated with release of effector cytokines (including IFN-γ, TNF-α and IL-2) that sculpts the local immune response to cancer (58). Primary CD8^+^ T cells isolated from the human blood and modified with chimeric antigen receptors (CAR) to express high affinity to tumor antigens have been large used in the clinical practice to treat patients with hematological malignancies (59, 60). However, CD8^+^ T cell exhaustion is a major limitation to the efficacy of cytotoxic anti-tumor responses, particularly limiting the application of CAR T cells to solid tumors, due to persistent TCR activation in the tumor microenvironment (61). To overcome exhaustion in primary CD8^+^ T cells and CAR T cells, combination therapy with ICI has emerged as an attractive strategy for increasing efficacy (62, 63). Whereas CAR T cell therapy targeting CD8^+^ T cells to tumor antigen has shown remarkable efficacy for treating patients with certain B cell driven hematological malignancies (64), ICI has significantly enhanced life-expectation in patients with a broad range of solid tumors (65–68) and became the first-line therapy for advanced melanoma patients, given its improved clinical efficacy and improved safety profile in comparison with conventional cancer therapies (65).

### 4.2 Conventional CD4^+^ T cells

CD4+ T cells have largely been neglected because most tumors lack MHC II expression and cannot directly be recognized by these T cells. However, CD4^+^ T cells play helper functions through secretion of cytokines that orchestrate the immune response and have a dual role in the tumor microenvironment. They can destroy the tumor vasculature, induce cellular senescence of cancer cells, and help CD8^+^ T cells in the effector phase, a role mostly associated with T-helper (Th)1 responses mediated by IL-2 and IFN-γ producing CD4^+^ T cells (69). Besides Th1 cells, IL-17-producing Th17 cells may also have potent anti-tumor immune effects by recruiting immune cells into tumors, activating effector CD8^+^ T cells, or even directly by converting toward Th1 phenotype and producing IFN-γ (70, 71). Th2 cells have also been shown to destroy tumor cells by inducing necrosis (72) and the therapeutic effectiveness of Th2 CD4^+^ CAR T cells has been demonstrated in a preclinical model of myeloma (73). Contrarily to these effects, Th2 and Th17 CD4^+^ T cells are also thought to have pro-tumorigenic activities mainly involving induction of cytokines that can promote growth, proliferation, and invasion of tumor cells, including IL-4 and IL-17, respectively (74–76).

### 4.3 Treg cells

These are an immunosuppressive subset of CD4^+^ T cells characterized by the constitutive expression of the transcription factor forkhead box protein 3 (FoxP3) and present essential roles in maintaining central tolerance. FoxP3^+^ natural Treg cells are generated in the thymus as a functionally mature T cell subpopulation specialized in immune suppression, hindering immunosurveillance against cancer development and hampering effective anti-tumor immune responses (77, 78).

Treg cells exert their immunosuppressive effects through various cellular and humoral mechanisms: (i) consumption of IL-2, thus inhibiting the proliferation and differentiation of conventional T cells (79), (ii) production of the inhibitory immune cytokines, IL-10 and TGF-β (80) and (iii) high expression of CTLA-4, which induces suppression of antigen-presenting cells (81). Treg expansion is associated with induction of c-Fos and elevated transcription of FoxP3 (82). FoxP3^+^ Treg cells with an activated phenotype can be enriched in tumors in comparison with peripheral blood and are associated with a poor prognosis in patients with various types of cancer, including cervical, renal, melanomas, and breast cancers (83, 84). Inversely, Tregs have also been associated with improved survival in colorectal, head and neck, and esophageal cancers (85, 86). This apparent paradoxical role of Treg may be associated with the fact that most of the studies rely on the solely detection of FoxP3 expression, which is transiently increased in activated conventional CD4^+^ T cells and cloud the specific identification of Treg (87, 88).

### 4.4 CD4^+^CD8^+^ double positive (DP) T cells

Classically, DP T cells are considered as a developmental stage in the thymus, before maturation as either CD4^+^ or CD8^+^ SP cells, but have been described in the peripheral blood and tissues in various settings, including in human cancers and some infectious diseases (89–91). The role of DP T cells in the periphery remains largely understudied, and it is unclear whether these cells escape the thymus in their immature stage expressing both co-receptors or if they originate from mature SP thymocytes that re-express the opposite co-receptor. DP T cells have been described in blood of patients with melanoma, bladder, prostate, kidney cancers (89, 92). The conflicting literature regarding the role of DP T cells — cytotoxic vs. immunosuppressive (93) — may indicate that these cells are heterogeneous and/or show pleiotropic functions that need to be investigated in each disease context. DP T cells favor the polarization of naïve CD4^+^ T cells into a Th2 functional profile (89). This previously unrecognized capacity of DP T cells was observed in healthy donors and exacerbated in patients with urologic cancer, who also showed elevated levels of circulating DP T cells (89).

### 4.5 Mucosal-associated invariant T (MAIT) cells

These are a class of innate-like T cells that exists in a pre-primed memory state and express a semi-invariant TCR that recognizes non-peptide antigens presented by the non-polymorphic MHC class I-like molecule, MRI (94). MAIT cells express CD8 in humans and either CD4 or CD8 in mice (95). Due to their multiple functions often associated with successful anti-tumor immune responses, MAIT cells represent an attractive population to explore for their potential roles in anti-tumor immunity (96). However, these cells represent a controversial topic in the field of tumor immunology with studies showing conflicting results regarding as to whether they contribute to tumor growth, tumor regression, or play a neutral role in human cancers (97, 98). Once activated, tumor-infiltrating MAIT cells display decreased IFN-γ and TNF-α and increase IL-17 production. Early investigations of MAIT cells in cancer suggested they may represent a potential positive prognostic marker. Indeed, a study screening of ~18,000 human tumors across 39 malignancies found a significant association of the KLRB1 gene (encoding CD161, a marker of MAIT cells), indicating a favorable prognosis (99).

### 4.6 Natural killer T (NKT) cells

These are a small population of true thymus-dependent T cells which are distinct from conventional T cells. Their TCR recognizes lipids rather than peptides and is restricted by a non-classical class I-like (class Ib) molecule CD1d (100, 101). Both type I and type II NKT cells play critical roles in tumor immunity; most often type I NKT cells promote anti-tumor immunity and type II NKT cells suppress it (102, 103). Moreover, both type I and II NKT cells have a myriad of interactions with other immune effector and regulatory cells, forming a complex web of immune regulation.

Type I and II NKT cells can cross-regulate each other, forming an immunoregulatory circuit that comes into play in the early steps of immune responses (104). When type I NKT cells are absent, both Tregs and type II NKT cells can exert suppressive activity upon the same tumor, and this situation mimics that often found in cancer patients, in which type I NKT cells are deficient in numbers or function. The role of type I NKT cells in protection against cancer has been found to be largely dependent on production of Th1 cytokines, especially IFNγ, even though NKT cells have lytic activity and could potentially directly lyse tumors expressing CD1d (102, 105).

### 4.7 TCRγδ T cells

These are the non-classical thymus-derived cell subgroup characterized by expression of γδ heterodimeric T cell receptor (TCRγδ) on cell surface, playing important roles in tumor immunity. Depending on the microenvironment, different γδ T cell subsets can have anti-tumor or pro-tumor activities (106). TCRγδ T cells can enhance the anti-tumor ability of other immune cells by secreting cytokines or expressing costimulatory molecules (107). Accordingly, this cell population has been safely used in clinics for the treatment of NSCLC and breast cancer (108). γδ gd T cell-based immunotherapy appeared to be safe and well-tolerated in patients (109, 110). However, TCRγδ T cells represent one of the main source of IL-17 in the tumor microenvironment, promoting ovarian cancer and pancreatic cancer progression (111, 112). In these studies infiltrating TCRγδ T cells could also directly induce the apoptosis of anti-tumor immune cells (112) at the same time they have shown to promote tumor development and metastasis by enhancing angiogenesis and recruiting inhibitory cells to the tumor site (113). Thus, it urges that the mechanisms controlling the anti-tumor versus pro-tumor activity by this cell type must be clarified, so that to better design therapeutic strategies targeting TCRγδ T cells.

In summary, unconventional T cells differ from their conventional counterparts in the rapidity of their initial response, the way they recognize and respond to nonpeptidic molecules, as well as their tissue distribution within the body (114). As the complexities of the immune system continue to be elucidated, it has become increasingly apparent that conventional and unconventional T cells operate in fundamentally different ways to mediate and coordinate host’s immune response to tumor cells.

## 5 Thymic cancer and its impact on T cell development

Thymomas are rare neoplasms of the thymic epithelial cells and present several abnormalities that may affect normal T cell development (91). Theories attempting to explain the association between autoimmune disorders and thymomas are based on the failure of positive and negative selection of thymocytes, the absence of regulatory mechanisms provided by AIRE, and on a Treg-poor environment in the neoplastic thymus (115). Myasthenia gravis is the most common disorder associated with thymoma, often linked to T cell-mediated autoimmunity in 30% of patients with thymoma (116, 117). Pioneer work screening tumors excised from patients with thymomas revealed their endocrine contents and MHC molecules, evidencing that thymoma epithelial cells contained large amounts of thymulin, thymosin α 1 and thymopoietin (48) and did not express the MHC class II-encoded molecules, HLA II-DR and -DC (117). These were the first studies showing that thymoma epithelial cells are endocrinologically active but present a defective antigen-presenting cell function, a potential mechanism for thymoma-associated autoimmunity. The finding that immunosurveillance towards cancer cells may be impaired before the diagnosis of thymoma (118) may challenge current theories attempting to explain immune disorders in patients with thymoma, suggesting that some immune events may precede the thymoma itself. It is likely that a combination of mechanisms, yet to be elucidated, is responsible for immune disorders in patients with thymoma. Again, further studies are crucial for design therapeutic alternatives to this cancer.

## 6 Manipulation of the thymic microenvironment to support anti-tumor immunity

Strategies aiming to protect the thymus are required to decrease side-effects of cyto-ablative cancer therapies (**Figure 1A**). Cytokines and soluble mediators secreted by the thymic microenvironment can impact prognosis of patients suffering from multiple types of cancer and have been manipulated to promote thymus regeneration, as reviewed below:

### 6.1 IL-22

Although IL-22 is not required for the formation or maintenance of the thymus under steady-state physiological conditions, it is produced intrathymically by T cells and innate lymphoid cells, presenting a paracrine role in driving thymic regeneration following radiation damage through induction of TEC survival and proliferation (119). Although the regenerative function of IL-22 is beneficial, its expression is confined to a period of tissue repair and persistence in the microenvironment helps tumors to escape cell cycle control and eradication by cytotoxic drugs. IL-22 is increased in the tumor of patients with non-small cell lung cancer (NSCLC), pancreatic cancer, gastric cancer and hepatocellular carcinoma and predicts a poor prognosis, higher disease stage, and faster tumor progression (120–123). Tumor cells from both murine and human lungs promote IL-22 production by memory T cells *via* induction of IL-1 (124). Expression of its cognate receptor, IL-22R1, is restricted to the non-hematopoietic cells, which makes the IL-22-IL-22R1 pathway an attractive target for cancer therapy.

### 6.2 Bone morphogenetic protein 4 (BMP4)

It is produced by thymic endothelial cells in response to radiation, and acts as a regulator of thymic regeneration after acute injury due to its role in stimulating bipotent TEC progenitors (TEPC) present in the adult thymus (125, 126). Accordingly, certain chemotherapeutic drugs reduce production of BMP4 and further damage the thymus (125, 126). BMP signalling has both tumor-promoting as well as -suppressing effects: at the same time that BMP4 is an important regulator of cell migration and invasion and induces epithelial–mesenchymal transition (EMT), an event that is crucial for the ability of cancer cells to acquire mobility and eventually metastasize, BMP4 can promote anti-tumor effects (127). TGFβ-mediated inhibition of BMP4 has been reported to promote breast cancer stem cell self-renewal activity, response to chemotherapy and is a good prognostic marker for patients with triple negative breast cancer (128). In mice, BMP4 induces differentiation of colorectal cancer stem cells and increases their response to chemotherapy (129). High expression of BMP4 in serous ovarian cancer is an independent prognostic factor for longer progression-free survival and overall survival (130). Contrarily to breast, colorectal and ovarian cancer, high expression of BMP4 in hepatocellular carcinoma promotes tumor progression (131).

### 6.3 Keratinocyte growth factor (KGF)

Keratinocyte growth factor (KGF) is a member of the fibroblast growth factor family mostly produced by cells of mesenchymal origin and plays an important role in protecting and repairing epithelial tissues. In the thymus, mesenchymal cells (fibroblasts) enhance the proliferation of TEC *via* the production of KGF during fetal development (132). In the adult thymus, KGF is produced by mature thymocytes, which mediates thymic epithelial cell proliferation and differentiation (133). KGF knockout mice are more vulnerable to sublethal irradiation, and endogenous administration of KGF attenuates the negative effects of acute thymic injury caused by chemotherapy and irradiation in middle-aged mice (134). In a phase III trial involving patients with hematologic malignancies who were treated with chemoradiotherapy before autologous peripheral blood progenitor cell adoptive transfer, recombinant human KGF (palifermin) treatment significantly reduced both the incidence and duration of severe oral mucositis (135). These data suggest that KGF can be used as a strategy to decrease adverse effects associated with cancer therapies.

### 6.4 RANK ligand (RANKL)

It is a TNF family member produced intrathymically by positively selected thymocytes and lymphoid tissue inducer cells (136). RANKL binds to its cognate receptor activator of nuclear factor kappa-B (RANK) expressed on the surface of mTEC to induce cellular expansion and differentiation into AIRE^+^ cells, establishing central tolerance (137). Clinically, increase in serum RANKL levels is associated with incidence of breast cancer in postmenopausal women (138). RANKL inhibition with Denosumab, a fully humanized antibody, improves bone-metastasis free survival in patients with breast cancer, prostate cancer and other solid tumors, an effect known to be associated with prevention of RANKL-RANK signaling on osteoclasts (139–141). A recent study using a poorly immunogenic murine melanoma model show that transient RANKL blockade interrupt central tolerance and unleashes T cells possessing immature TCR to recognize tumor self-antigens and improve response to immunotherapy (142). These data evidence that the therapeutic value of blocking the RANKL/RANK axis for cancer therapy is both due to its direct action in preventing skeletal-related adverse effect and in shaping intrathymic T cell development.

### 6.5 Interruption of thymic activity

Therapeutical thymectomy for thymic epithelial tumors has been an established procedure for more than 40 years and is associated with several paraneoplastic autoimmune syndromes due to a loss of central tolerance (143, 144). Although it is assumed that thymectomy renders immunosuppression due to disturbance in the pool of conventional T cells in the periphery, a recent study conducted in a preclinical model of primary melanoma documented that cessation of thymic activity in adult mice causes preferential reduction of Treg exports to the periphery, thus increasing the efficacy of anti-tumor immunotherapies targeting the immune checkpoint inhibitor CTLA-4 (145). Corroborating these findings, therapeutical thymectomy for thymoma prevents the increase of Treg cells in the circulation following immunosuppressive therapy (146).

Apart from its role in Treg export, interruption of thymus activity by genotoxic chemotherapy induces secretion of molecules by the thymic microenvironment and creates a chemoprotective niche harboring surviving lymphoma cells following chemotherapy (147). In support to these findings, there is evidence that tumor cells can hide in the thymus and acquire chemo-resistance. The presence of cancer cells within the thymus has been demonstrated in mice injected with 3LL lung tumors (148), while athymic mice presented significantly fewer chemo-resistant lymphoma cells and lived considerably longer than immunocompetent mice (147).

These data are from pre-clinical experimental models, and future studies in humans will provide more information on the impact of thymectomy in anti-tumor immunity and response to chemotherapy.

## 7 Efficacy of thymic peptides in clinical and preclinical cancer research

Purified thymus extracts are thought to enhance the immune system of patients with cancer, promoting elimination of tumor cells and resistance to opportunistic infections, which are often associated with the use of conventional therapies for cancer (9). Derivatives of thymic peptides, mostly of thymosins, have been detected as products of neoplastically transformed TEC and employed in the early diagnosis and treatment of neoplasms. Besides, studies in animal models and human patients have shown promising results in different types of malignancies, especially when Tα1 was used in combination with other cancer therapies, as reviewed below:

### 7.1 Clinical use of Tα1 in solid tumors

The therapeutic use of the Tα1 synthetic analogue, thymalfasin, for treating several diseases is currently approved in over 35 countries worldwide for its immunomodulatory activities and safety (10). Approved indications for medical use include adjuvant to chemotherapy. Combination of Tα1 with chemotherapy has been of value to treat oncologic patients, with reference to melanoma, non-small cell lung cancer (NSCLC) and hepatocellular carcinoma.

#### 7.1.1 Melanoma

Dacarbazine (DTIC) is considered the benchmark treatment for advanced melanoma, despite response rates of less than 10% in contemporary trials. A phase II study analyzing the effect of combination of DTIC+Tα1+IL-2 in treating patients with metastatic melanoma showed objective responses in 36% of the patients analyzed and no safety concerns (149). A following up study aiming to analyze the effect of DTIC+Tα1+low dose IFN-α in treating melanoma patients demonstrated response rate of 50%, associated with increase in CD4^+^ T cells and NK cell numbers in the peripheral blood (150). Due to the low cohort size and absence of Tα1 monotherapy arm for direct comparison, both studies failed to demonstrate a survival benefit for patients receiving Tα1. In larger following up randomized trial involving 488 patients with metastatic melanoma randomly assigned to 5 groups including DTIC+Tα1+IFN-α; DTIC+Tα1; DTIC+IFN-α (control group), 10 and 12 tumor responses were observed in the DTIC+IFN-α+Tα1 and DTIC+Tα1 groups, respectively, versus 4 in the control. Response rates ranged from 1.9 to 23.2 months in patients given Tα1 and from 4.4 to 8.4 months in the control group (151). The high rates of stable disease (26% to 37%) observed in patients treated with Tα1 are characteristic of immunotherapy, where the decline in tumor volume tends to occur slowly and progressively with continued treatment (152). Although the mechanism underlying the activity of DTIC+Tα1 is not fully understood, it is possible that Tα1 potentiates T cell–mediated immune responses directed against tumor antigens.

#### 7.1.2 NSCLC

Tα1 has been used with chemotherapy to treat NSCLC. A study performing a systematic review and meta-analysis of 27 randomized controlled trials in China containing 1925 patients with NSCLC demonstrated clinical efficacy and safety of combination therapy with synthetic thymic peptides and chemotherapy. Optimal conditions for Tα1 treatment included combination with gemcitabine or navelbine and cisplatin, twice a week, with one 3-week cycle (153). In another retrospective study, 5746 patients with margin-free-resected NSCLC patients were divided into the Tα1 group and the control group according to whether Tα1 was used or not after surgery (154). The 5-year disease-free survival and overall survival rates were significantly higher in the Tα1 group compared with the control group (77.3% versus 57.9% and 83.3% versus 65.6%, respectively). This was observed in all subgroups of age, sex, smoking status, and pathological tumor-node-metastasis stage, especially for patients with non-squamous cell NSCLC and without targeted therapy (154). More recently, phase 2 trial where 69 patients received Tα1 during and after chemoradiotherapy based on docetaxel and nedaplatin demonstrated significant reductions in radiation-induced pneumonitis and lymphopenia compared with control group (36.2% versus 53.6% and 19.1% versus 62.1%, respectively) (153).

#### 7.1.3 Hepatocellular carcinoma

In a small phase II randomized trial for unresectable hepatocellular carcinoma where 25 patients were enrolled, Tα1 administration to patients that have gone through transarterial chemoembolization (a combination of regional chemotherapy and some form of hepatic artery occlusion) 5 times weekly for 24 weeks resulted in numerically higher rates (although not statistically significant) of tumor response (155). Through 72 weeks, 57.1% (8/14) of patients in the group receiving TACE + thymalfasin became responders to cytoablative therapy versus 45.5% (5/11) in the group receiving TACE only. Among the 8 responders in the group receiving TACE + thymalfasin, 4 patients became eligible for liver transplant whereas none of the 5 responders in the TACE-only group became eligible for transplant. A larger study enrolling a total of 206 patients with small hepatocellular carcinoma who received liver resections to evaluate the effect of Tα1 as an adjuvant therapy demonstrated a statistically significant increase in 5-years overall survival and recurrence-free survival (82.9% versus 62.9% and 53.3% versus 32.1%, respectively) for patients that received Tα1 in comparison to those that went through resection only (156). Therefore, Tα1 as an adjuvant therapy may improve the prognosis of hepatocellular carcinoma patients.

### 7.2 Preclinical studies on thymic hormone using murine models

The first observations that Tα1 could play a protective role in melanoma came from the work published in 1983 showing that Tα1 was able to protect mice immunosuppressed with 5-flurouracil chemotherapy from infection by opportunistic pathogens (157). In two other publications from the same year the group showed that Tα1 could similarly protect mice immunosuppressed with cytostatics or X-ray irradiation from metastatic growth and increase survival (30, 31). A more recent study showed that combination of Tα1 with cyclophosphamide significantly increased the median survival time of treated mice, and cured an average of 23% of animals, while none was cured in mice treated with cyclophosphamide only. This was associated with increase of T cell numbers, expression of IL-2 receptor and cytotoxic responses (158). The relevance of IL-2 in potentiating anti-tumor activity of Tα1 in combination with cyclophosphamide was further evidenced in mouse model of Lewis lung carcinoma, where depletion of T cells abolished the positive response to combination therapy (159). Further, chemo-immunotherapy with 5-fuorouracil (5-FU)+Tα1+IL-2 had superior activity over all treatments tested as monotherapies in preventing liver metastases in a rat colorectal cancer model (160). To improve Tα1 targeting of tumor cells, Tα1 was combined with RGD (Arg-Gly-Asp), which has been utilized in delivering anticancer drugs to tumor sites. Results showed that Tα1-RGD had remarkable anti-tumor effects, and its tumor targeting was better than that of Tα1 (161).

Further, several lines of evidence converge to the notion that Tα1 represents a plausible candidate to improve the safety of ICI. This is evidenced in a murine model of ICI-induced colitis where Tα1 administration prevented intestinal toxicity by promoting the indoleamine 2,3-dioxygenase (IDO) 1-dependent tolerogenic immune pathway (162). Despite improving safety of ICI, Tα1 monotherapy showed clear anti-metastatic benefit in a mouse melanoma lung metastasis model but no increase in effectiveness was observed upon addition of anti-PD1 (163).

Altogether, these results provide direct evidence that Tα1 can significantly affect tumor development in humans and murine models. As mentioned above, contrasting with Tα1, thymopoietin expression positively correlates with tumor development (30, 31). In this respect, it is conceivable that neutralizing thymopoietin expression might bring therapeutic advances. As regards thymulin, despite its anti-inflammatory activity well defined (51, 164), data are still lacking in terms of its potential modulatory role on cancer, and to our knowledge no clinical trials have been engaged on this aspect.

## 6 Conclusion and future direction

We have briefly reviewed some of the potential impacts of thymic-mediated immune and endocrine effects on modulating cancer evolution, emphasizing the relevance of future studies aiming to improve cancer prognosis and reducing side effects of treatments through manipulation of the thymus.

Since thymic-derived immune cells, hormones and cytokines can circulate in the blood impacting diverse aspects of the host’s immune system and tumor cell biology, it is important to understand the immunoendocrine interactions during oncogenic diseases in humans and in preclinical models. Further, the possibility of purifying and manipulating thymic peptides and immature T cells able to recognize tumor self-antigens for clinical use, makes the thymus an attractive target for cancer therapy.

## Author contributions

AL, conceptualization and writing the original draft. WS, visualization and proofreading. All authors contributed to the article and approved the submitted version.
